# Self-Nanoemulsifying Drug Delivery System of 2-Methoxyestradiol Exhibits Enhanced Anti-Proliferative and Pro-Apoptotic Activities in MCF-7 Breast Cancer Cells

**DOI:** 10.3390/life12091369

**Published:** 2022-09-02

**Authors:** Salwa D. Al-Qahtani, Hawazen H. Bin-Melaih, Eman M. Atiya, Usama A. Fahmy, Lenah S. Binmahfouz, Thikryat Neamatallah, Fahad A. Al-Abbasi, Ashraf B. Abdel-Naim

**Affiliations:** 1 Department of Medical Laboratory Sciences, Faculty of Applied Medical Science, Majmaah University, Majmaah 11952, Saudi Arabia; sd.alqahtani@mu.edu.sa; 2 Department of Biochemistry, Faculty of Science, King Abdulaziz University, Jeddah 21589, Saudi Arabia; ehusseinatiya@stu.kau.edu.sa (E.M.A.); fabbasi@kau.edu.sa (F.A.A.-A.); 3 Department of Biological Sciences, Faculty of Sciences, King Abdulaziz University, Jeddah 21589, Saudi Arabia; habdullahbinmelaih@stu.kau.edu.sa; 4 Department of Pharmaceutics, Faculty of Pharmacy, King Abdulaziz University, Jeddah 21589, Saudi Arabia; uahmedkauedu.sa@kau.edu.sa; 5 Department of Pharmacology and Toxicology, Faculty of Pharmacy, King Abdulaziz University, Jeddah 21589, Saudi Arabia; lbinmahfouz@kau.edu.sa (L.S.B.); taneamatallah@kau.edu.sa (T.N.)

**Keywords:** 2-methoxyestradiol, breast cancer cells, self-nanoemulsifying drug delivery system, cytotoxicity, apoptosis

## Abstract

(1) Background: 2-Methoxyestradiol (2ME) is a metabolite of estrogens and possesses promising anti-proliferative and cytotoxic activities. However, it suffers unfavorable pharmacokinetic characteristics such as absorption after oral administration. The aim of this study was to prepare an optimized 2ME self-nanoemulsifying drug delivery system (2ME-SNEDDS) and evaluate its cytotoxicity and pro-apoptotic activities in MCF-7 breast cancer cells. (2) Methods: For optimization of the 2ME-SNEDDS, a three-component system was used in the D-optimal mixture experimental study. MCF-7 cells were incubated with the 2ME-SNEDDS and subjected to an assessment of growth inhibition, cell cycle progression, annexin V staining, caspase-3 concentration, Bax, Bcl-2, and cyclin D1 mRNA expression, and reactive oxygen species (ROS) generation. (3) Results: The optimized formula had a globule size of 94.97 ± 4.35 nm. Zeta potential was found to be −3.4 ± 1.2 mV with a polydispersity index (PDI) of 0.34. In addition, 96.3  ± 4.3% of 2ME was released from the 2ME-SNEDDS within 24 h using the activated analysis bag technique. Moreover, the prepared 2ME-SNEDDS exhibited a significant enhancement of the anti-proliferative activity against MCF-7 cells in comparison to raw 2ME. This was associated with cyclin D1 expression down-regulation and the accumulation of cells in the G0/G1 and G2/M phases. The pro-apoptotic activities of the 2ME-SNEDDS were confirmed by annexin V staining, which indicated enhanced early and late cell death. This accompanied modulation of the mRNA expression of Bax and Bcl-2 in favor of apoptosis. The 2ME-SNEDDS significantly enhanced cleaved caspase-3 concentration in comparison to raw 2ME. In addition, the 2ME-SNEDDS significantly increased the generation of ROS in MCF-7 cells. (4) Conclusions: The 2ME-SNEDDS exhibits enhanced cytotoxicity and pro-apoptotic activity in MCF-7 cells. This is mediated by, at least partially, ROS generation.

## 1. Introduction

Global statistics show that breast cancer is the most frequently diagnosed female malignancy and is the fifth leading cause of death from cancer [1]. The lifetime risk of developing breast cancer is one female in eight [2]. The incidence of breast cancer in developing countries continues to increase; however, the mortality rate has declined considerably [3]. This is attributed to both advanced treatment approaches and screening systems’ implementation and advanced imaging techniques [4]. Unfortunately, cancer chemotherapy suffers resistance and severe drug-induced toxicity. Therefore, there is an urgent need for new effective and safe drugs [5]. 

2-Methoxyestradiol (2ME), a metabolite of 17β-estradiol, is a natural compound and a hormone that is produced by both males and females. The metabolism of endogenous estradiol primarily involves cytochrome P450 (CYP)-dependent hydroxylations at C-2, -4, or -16, yielding either 2- or 4-hydroxyestradiol estrogens, or 16a-hydroxyestradiol. Catechol-O-methyltransferase (COMT) is involved in catalyzing the metabolism of 2-hydroxyestradiol to form 2ME. During pregnancy, its physiological level in the blood ranges from 30 pM to 30 nM [6], whereas pharmacologically relevant concentrations involve micromolar concentrations [7]. The induction of oxidative stress plays a role in 2ME’s anti-tumor activity against various cancer cellular models [8]. Moreover, 2ME, is an anti-angiogenic and anti-proliferative molecule that effectively induces apoptosis. The effectiveness of 2ME has been demonstrated against different types of cancer cells [9]. 2ME alone or combined with other drugs has been escalated to clinical trials to treat different tumors with high metastatic potential [7,10,11]. 2ME does not possess estrogenic activity due to its weak affinity to estrogen receptors [12]. Several mechanisms for 2ME activity have been proposed, including effects on tubulin polymerization and depolymerization, as well as the induction of mitochondrial apoptotic signaling [13]. However, 2ME suffers unfavorable pharmacokinetic characteristics such as absorption after oral administration and poor cellular permeability [14,15]. 

The use of nanotechnology in cancer treatment has experienced exponential growth in the last years [16]. In particular, a lipid-based emulsion system has emerged as an appealing option to improve the solubility of water-insoluble molecules. Additionally, these systems improve cellular permeability and consequently enhance pharmacokinetic properties [17]. Self-nanoemulsifying delivery drug systems (SNEDDSs) are mixtures of oil, surfactant, and co-surfactant that offer several advantages over raw drugs including enhanced solubility and cellular permeability [18]. In addition, SNEDDSs have been used to enhance the pharmacological activities of other molecules [19,20,21,22]. Therefore, the aim of this study was to prepare an optimized 2ME-SNEDDS and evaluate its cytotoxicity and pro-apoptotic activities in MCF-7 breast cancer cells.

## 2. Materials and Methods

### 2.1. Chemicals and Media

2-Methoxyestradiol (2ME) was purchased from Fraken Biochem Co., Ltd., (Qingdao, China) with purity more than 98%; ethanol, chloroform, and dimethyl sulfoxide (DMSO) were obtained from Sigma-Aldrich (St. Louis, MO, USA). Tween 80, cumin oil, and propylene glycol were purchased from Sigma-Aldrich (St. Louis, MO, USA). Thiazolyl Blue Tetrazolium Bromide (MTT) was obtained from Sigma-Aldrich Inc. (St. Louis, MO, USA). Eagle’s Minimal Essential Medium (EMEM) was obtained from Sigma-Aldrich Co. (St. Louis, MO, USA); TrypLE™ Express Enzyme (1X), phosphate-buffered saline (PBS), penicillin/streptomycin, and fetal bovine serum (FBS, Qualified) were purchased from Gibco (Grand Island, NY, USA). Doxorubicin (DOX) was purchased from MCE (Med Chem Express, Monmouth Junction, South Brunswick, NJ, USA). 

### 2.2. Solubility Study 

#### 2.2.1. D-Optimal Mixture Experimental Design for Formulation and Optimization of 2ME-SNEDDS

A three-component system was used in the D-optimal mixture experimental study: the oil phase X1 (cumin oil), the surfactant X2 (Tween 80), and the co-surfactant X3 (propylene glycol). Based on preliminary phase diagram studies, the following component ranges were chosen: X1 (15–30%), X2 (25–40%), and X3 (30–50%). As a response, the mean droplet size (Y) was used (dependent variable). Design-Expert^®^ software was used to analyze the responses of all formulations (version 11; Stat-Ease, Inc., Minneapolis, MN, USA). Several statistical parameters, including the standard deviation (SD), the multiple correlation coefficient (R2), the adjusted multiple correlation coefficient (adjusted R2), and the predicted residual sum of squares, were compared to determine the best fitting mathematical model (PRESS). Among them, PRESS indicates how well the model fits the data, and it should be small for the chosen model in comparison to the other models under consideration, while correlation coefficients should be maximum and close to each other. As a base design, the software selected a set of candidate points. Factorial points were among them (centers of edges, high and low level from the constraints on each factor, axial check point, constraint plane centroids, and an overall center point). The base design included 16 runs (Table 1). This study’s optimal formulation aimed at attaining the smallest possible droplet size as a basis for enhancing cell permeation.

#### 2.2.2. Droplet Size Measurement

One ml of 2ME-SNEDDS was diluted with 20 mL of deionized water and magnetically stirred before using a Malvern Zetasizer Nano ZS particle size analyzer to determine vesicle size (Malvern Instrument, Worcestershire, UK). The results were calculated as the mean of six determinations. 

#### 2.2.3. Optimization of 2ME-SNEDDS

Composition of the formulated 2ME-SNEDDS was optimized using numerical methods based on the desirability concept, whereby the optimization procedure was intended to reduce the size of SNEDDS. The concentrations of the researched components were projected for the modified compound, and the appropriateness factor was calculated. The modified formulation’s vesicle size, zeta potential, and polydispersity index (PDI) were determined as described above in Section 2.2.1.

#### 2.2.4. 2-ME-SNEDDS Release Assay

Briefly, an activated dialysis bag (MWCO = 12,000 Da) was placed in a dissolution chamber where the system was maintained at 100 rpm and 37 ± 0.5 °C. The release profile was determined in phosphate buffer (0.1 M pH 7.4). Samples of 3 mL volume were collected at specified time intervals and an equal quantity of fresh dissolution media were replenished in the assembly to maintain sink condition, which contained tween 80 (0.1%). 2ME-SNEDDS containing 2 mg 2ME were included in the experiment, as described. Samples were withdrawn at frequent intervals within 24 h and evaluated for 2ME concentration using a high-performance liquid chromatography method.

### 2.3. Cell Culture

MCF-7 (HTB-22) cells were purchased from the American Type Culture Collection (ATCC, Manassas, VA, USA). Cells were cultured in Eagle’s Minimal Essential Medium (EMEM) containing 10% (*v/v*) FBS (Sigma, St. Louis, MO, USA), 2 mM L-glutamine (Sigma), 20 mM HEPES (Sigma), 0.025 g/mL amphotericin B (Sigma), 100 IU/mL penicillin G (Sigma), and 100 g/mL streptomycin (Sigma) (Sigma). This study used cells between passages 10 and 20.

### 2.4. Cell Viability Assay

MTT ((3-(4,5-dimethylthiazol-2-yl)-2,5-diphenyltetrazolium bromide) assay was performed on human breast cancer cell line (MCF-7) to assess cell viability of V1 (dimethyl sulfoxide vehicle of raw 2ME), V2 (plain SNEDDS), raw 2ME, 2ME-SNEDDS, and doxorubicin (DOX). Cells were grown in Eagle’s Minimal Essential Medium (EMEM) supplemented with 100 units/mL penicillin, 100 μg/mL streptomycin, and 10% fetal bovine serum (FBS) at 37 °C, in a humidified, 5% CO2 atmosphere and split when they reached 80 to 90% cell confluence. Cells were seeded at a density of 5 × 10^3^ cells per well in 96-well plates and incubated overnight. The compounds were added to the cells at different concentrations ranging from 0.1 to 1000 µM followed by 48 h of incubation. At the end of incubation, the culture medium was aspirated and replaced with MTT solution at a final concentration of 0.5 mg/mL, followed by incubation for 3 h at 37 °C. After that, the MTT solution was removed, and 100 μL DMSO was added to dissolve the formazan crystals and incubated for 5 min. A microplate reader was used to measure absorbance at 570 nm. (Synergy HT, BioTek, Winooski, VT, USA). The following equation was used to calculate the percent cell viability

% Cell Viability = (Absorbance of treated cells/Absorbance of control untreated cells) × 100

### 2.5. Cell Cycle Distribution Assessment

Cell cycle was examined using propidium iodide (PI) flow cytometry kit for cell cycle analysis (Catalog #: ab139418, Abcam, Cambridge, UK). Cells were seeded at a density of 1 × 10^5^ cells/well in 6-well plates. At 24 h after incubation, the cells were treated to the predetermined IC50 values for 24 h. Cells were then trypsinized and centrifuged at 500× *g* for 5 min. After discarding the supernatant, the cells were washed, resuspended in 1 mL of 1X PBS, and centrifuged at 500× *g* for 5 min. The cells were fixed in ice-cold 70% ethanol and kept at 4 °C for 2 h. The cells were then washed twice with PBS. The supernatant was removed, and the pellet was washed with 1 mL of 1X PBS. Cells were centrifuged at 500× *g* for 5 min again, and the supernatant was removed. Cell pellets were stained with 200 mL of 1X propidium iodide + RNase staining solution and incubated in the dark for 20–30 min before being analyzed with the BD FACS Calibur and CellQuest software (BD Bioscience, San Jose, CA, USA). The FL2 was used to measure PI fluorescence (ex/em 488/636 nm).

### 2.6. Assessment of Annexin V Staining 

The effect of the compounds on apoptosis and necrosis cell death was determined using the annexin V-FITC Apoptosis Detection Kit (Catalog #: K101, BioVision Research Products, Mountain View, CA, USA) as directed by the manufacturer. MCF-7 cells were seeded at a density of 1 × 10^5^ cells/well in 6-well plates overnight. Following that, cells were exposed for 24 h to the predetermined IC50 values of each treatment. At the end of the incubation period, cells were trypsinized and centrifuged at 10,000× *g* for 5 min. After that, the cells were washed with PBS and centrifuged. The supernatant was discarded, and the cells were resuspended in 500 μL of 1X binding buffer before being incubated at room temperature for 5 min in the dark with 5 μL of annexin V-FITC and 5 μL of propidium iodide. Cells were injected into a BD FACS Calibur system (BD Biosciences, San Diego, CA, USA) and analyzed for FITC and PI fluorescent signals using FL1 and FL2 signal detectors (ex/em 488/530 nm for FITC and ex/em 488/617 nm for PI, respectively). In total, 10,000 events were collected for each sample. Positive FITC and/or PI cells were counted using quadrant analysis and the CellQuest software (BD Bioscience, San Jose, CA, USA).

### 2.7. Measurement of Active Caspase-3

Assessment of human active caspase-3 was performed using solid phase sandwich enzyme-linked immunosorbent assay ELISA kit (Cat # KHO1091, Invitrogen, Carlsbad, CA, USA). MCF-7 cells were seeded and treated with a predetermined IC50 of V1, V2, raw 2ME, 2ME-SNEDDS, and DOX for 24 h. The cells were then centrifuged and washed twice with cold PBS. The supernatant was discarded, and the pellets were lysed for 30 min on ice with vortexing in extraction buffer. Lysates were centrifuged for 10 min at 4 °C at 13,000 rpm, and clear lysates were transferred to clean microcentrifuge tubes. An aliquot of 100 μL of standards and samples were added to the microtiter wells for the assay, which was incubated for 2 h. The solution was then aspirated and washed four times with 400 µL of washing buffer. The wells were then filled with 100 μL of caspase-3 and incubated for 1 h at room temperature. The solution was drawn from the wells and washed four times. The plate was then incubated for 30 min at room temperature in the dark with 100 μL of anti-rabbit IgG HRP working solution. Wells were washed four times before being incubated in the dark for 30 min with 100 μL of stabilized chromogen. Finally, 100 μL of stop solution was added to each well, and the absorbance was measured using a plate reader at 450 nm (Synergy HT, BioTek, Winooski, VT, USA).

### 2.8. Real-Time Polymerase Chain Reaction (RT-PCR) 

RT-PCR was used to determine the expression of Bax, Bcl-2, and cyclin D1. MCF-7 cells were treated for 24 h with a predetermined IC50 value of treatments. The cell fraction was used to extract RNA, which was then used to synthesize cDNA. The Gene Runner software was used to create primers for Bcl-2, Bax, and cyclin D1 (Table 2). The prepared samples were estimated for the expression in triplicate, and the samples were normalized with reference to β-actin [23,24]. 

### 2.9. Measurement of ROS Generation 

An ROS Detection Assay Kit (Cat. # K936-100; BioVision, Milpitas, CA, USA) was used to detect intracellular ROS production. Overnight, cells were plated in a 96-well plate at a density of 5 × 10^3^ cells/well. The cells were then washed with 100 μL of ROS assay buffer, and 100 μL of 1X ROS label was added to each well before incubating for 45 min at 37 °C in the dark. Following that, cells were treated for 24 h with a predetermined IC50 of treatments. The intensity of the fluorescence was then measured using a microplate reader (Synergy HT, BioTek, Winooski, VT, USA) at Ex/Em = 495/529 nm.

### 2.10. Statistical Analysis

The data are presented as mean ± standard deviation (SD). One-way analysis of variance (ANOVA) was used to determine statistical significance, followed by Tukey’s post hoc test. At *p*-value 0.05, differences between samples were considered statistically significant. GraphPad prism software version 8.0.2 for Windows was used for all analyses (GraphPad Software Inc., San Diego, CA, USA).

## 3. Results

### 3.1. Model Fit Statistics and Diagnostic Analysis

The D-optimal design was chosen because it minimizes the variance associated with the coefficient estimates in the model and thus provides the best estimate for the effect of the variables on the response (droplet size). Droplet sizes in the prepared SNEDDS ranged from 240 to 1268 nm. Table 3 shows the droplet size data that best fit the special quartic model based on its highest correlation (R2) and lowest PRESS. Furthermore, the predicted and adjusted R2 values were reasonably consistent, and the adequate precision value of 18.72 (greater than 4) confirmed that the model could be used to navigate the experimental design space.

**Figure 1:**
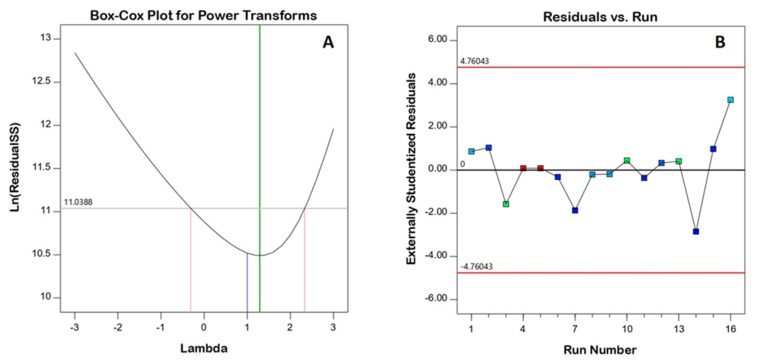
Diagnostic plots for droplet size of 2ME-SNEDDS; (**A**) Box–Cox plot and (**B**) predicted vs. actual values plot.

Figure 1 depicts diagnostic plots that assess the goodness of fit of the chosen model. Figure 1A shows a Box–Cox plot for power transforms with a best lambda (λ) value of 1.29 (represented by the green line). The computed confidence interval around this is −0.31–2.33 (represented by the red lines); the computed confidence interval includes the value 1 (currently represented by the blue line); thus, no specific data transformation is recommended [25]. Furthermore, the color points in the externally studentized residuals vs. the run number plot were distributed at random within the limits, indicating no constant error (Figure 1B).

### 3.2. Statistical Analysis of Droplet Size Data

**Figure 2:**
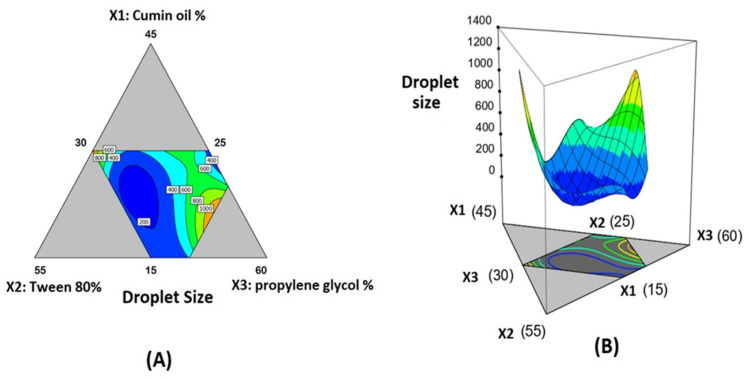
The effect of mixture components on the droplet size of 2ME-SNEDDS is depicted in 2D contour plot (**A**) and 3D surface plot (**B**).

ANOVA for droplet size confirmed that the special quartic model was significant, as evidenced by its F-value of 37.01 (*p* < 0.0001). The data fit the proposed model due to the non-significant lack of fit relative to pure error, with an F-value of 1.49 (*p* = 3.102). The statistical analysis revealed the importance of surfactant and co-surfactant proportions on droplet size, as well as their interaction (X_2_X_3_). A significant interaction among the three components were observed as indicated by the significant term X_1_X_2_X_3_^2^ (Table 4). Figure 2 also depicts contour and 3D surface diagrams demonstrating the effect of varying proportions of (X_1_), (X_2_), and (X_3_) on the droplet size of the SNEDDS. The three components of the mixture were located at the triangle vertices in the contour diagram (Figure 2A). The grey areas in the figure represent areas that were not used in the regression due to component constraints. The following equation was generated in terms of a coded factor to represent the special quartic model

Y = 6161.23X_1_ + 8426.25X_2_ + 4727.32X_3_ − 24,121.85X_1_X_2_ − 20,572.17X_1_X_3_ − 24,927.83X_2_X_3_ + 43,994.89X_1_^2^X_2_X_3_ − 24,722.60X_1_X_2_^2^X_3_ + 98,193.72X_1_X_2_X_3_^2^

#### 3.2.1. Optimization of 2ME-SNEDDS

Numerical optimization following a desirability approach was employed for the prediction of the optimized mixture components that could yield the lowest possible size. As per the optimization technique, the optimized levels of cumin oil, Tween 80, and PG percentages were 25.72%, 36.82%, and 37.46%, respectively. The measured size of the optimized formulation was 53 nm, which was in accordance with the predicted value of 94.97 ± 4.35 nm with an error percentage of 2.3%. The relatively low percentage error verifies the validity of the optimization process. Zeta potential was found to be −3.4 ± 1.2 mV with a PDI value of 0.34.

#### 3.2.2. Release of 2ME from the SNEDDS

**Figure 3:**
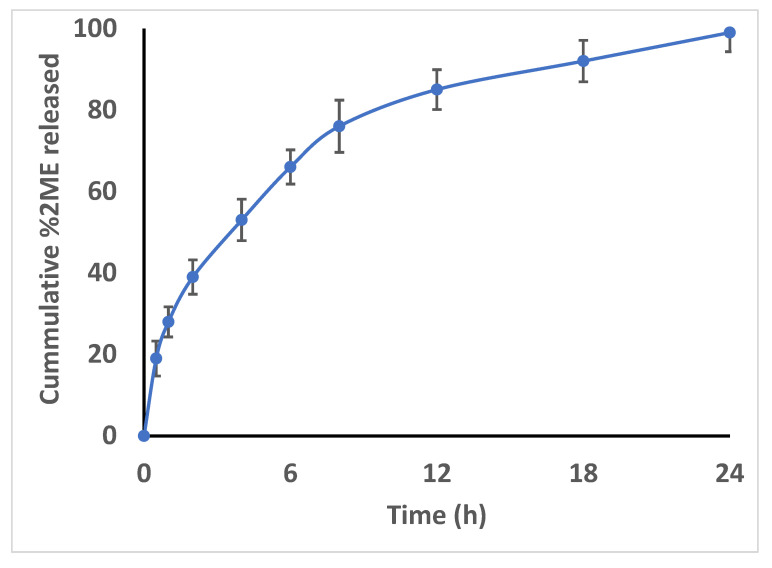
In vitro release pattern of optimized 2ME-SNEDDS after 24 h; activated dialysis bag method was used in a dissolution chamber where the system was maintained at 100 rpm and 37 ± 0.5 °C. The release profile was determined in phosphate buffer pH 7.4 (physiological pH) in sink condition containing 0.1% tween 80.

Analysis of the in vitro 2ME release from the formulated 2ME-SNEDDS demonstrated a complete release pattern. Figure 3 illustrates that 96.3  ± 4.3% of 2ME was released from the 2ME-SNEDDS within 24 h (Figure 3).

### 3.3. Assessment of Cytotoxicity

**Figure 4:**
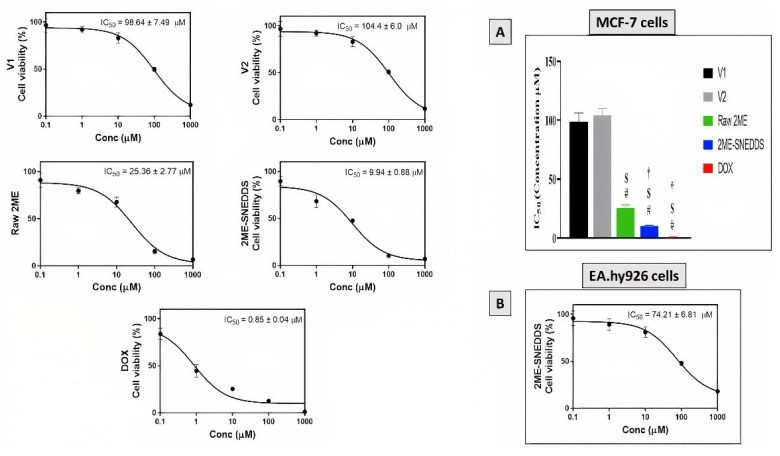
(**A**) Concentration–response curves of V1, V2, raw 2ME, 2ME-SNEDDS, and DOX in MCF-7 cells. (**B**) Concentration–response curve of 2ME-SNEDDS in EA.hy926 cells. Results are presented as mean ± SD (*n* = 6). # Significantly different from V1 at *p* < 0.05, $ significantly different from V2 at *p* < 0.05, † significantly different from raw 2ME at *p* < 0.05.

MTT assay was used to evaluate the cytotoxic activity of V1, V2, raw 2ME, 2ME-SNEDDS, and DOX against the MCF-7 cells after an incubation period of 48 h. The dose–response curves were used to calculate IC50 values as shown in Figure 4. The IC50 value for the raw 2ME was 25.36 ± 2.77 µM, whereas for the 2ME-SNEDDS it was 9.94 ± 0.88 µM. However, the 2ME-SNEDDS showed an approximate 60.8% decrease in the IC50 value compared to the raw 2ME (Figure 4A). Furthermore, the optimized 2ME-SNEDDS formula was tried in the normal human endothelium EA.hy926 cells and showed an IC50 value of 74.21 ± 6.81 µM (Figure 4B).

### 3.4. mRNA Expression of Cyclin D

**Figure 5:**
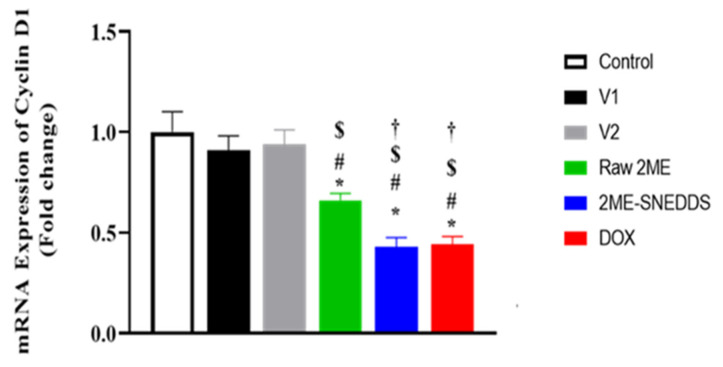
mRNA expressions of cyclin D1 in MCF-7 cells after incubation with V1, V2, raw 2ME, 2ME-SNEDDS, and DOX. Results are presented as mean ± SD (*n* = 6). * Significantly different from control at *p* < 0.05, # V1 at *p* < 0.05, $ significantly different from V2 at *p* < 0.05, † significantly different from raw 2ME at *p* < 0.05.

As can be seen in Figure 5, the mRNA expression of cyclin D1 was not statistically affected by the incubation of MCF-7 cells with either V1 or V2. However, both raw 2ME and the 2ME-SNEDDS significantly down-regulated it to almost one-third of that observed in control incubations.

### 3.5. Analysis of Cell Cycle

**Figure 6:**
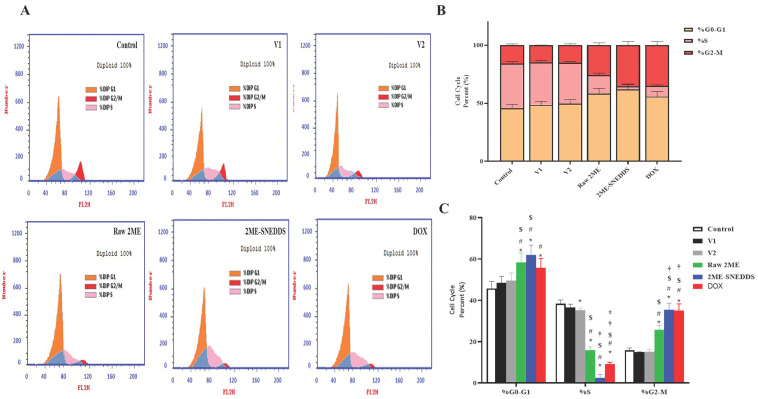
Flow cytometric analysis of cell cycle distribution of PI-stained MCF-7 cells. (**A**) Representative flow cytometry histograms after treatment with V1, V2, raw 2ME, 2ME-SNEDDS, and DOX. (**B**) Cumulative bar chart showing the percentage of cells in the G0/G1, S, and G2/M phases of the cell cycle. (**C**) Graphic presentation of the percentages of cell cycle phases. Results are presented as mean ± SD (*n* = 6). * Significantly different from control at *p* < 0.05, # significantly different from V1 at *p* < 0.05, $ significantly different from V2 at *p* < 0.05, † significantly different from raw 2ME at *p* < 0.05, ‡ significantly different from 2ME-SNEDDS at *p* < 0.05.

Analysis of the cell cycle of MCF-7 cells incubated with V1, V2, raw 2ME, 2ME-SNEDDS, and DOX is displayed in Figure 6A–C. V1 and V2 did not exhibit any change in the percentage of cells in the different stages of the cell cycle as compared with the control cells. Treatment of the MCF-7 cells with raw 2ME resulted in an increase in the cells in the G0/G1 phase and G2/M phase (58.42% and 25.79%, respectively) and a corresponding decrease in the S phase (15.79%). Additionally, the 2ME-SNEDDS showed a significant increase in the G0/G1 phase and G2/M phase (62% and 35.46%, respectively) and a significant decrease in the S phase (2.54%). It is worth mentioning that the 2ME-SNEDDS exhibited significantly more potent activities in inhibiting accumulating cells in the S phase and enhancing the portion of cells in the G2/M phase as compared to raw 2ME.

### 3.6. Annexin V Staining

**Figure 7:**
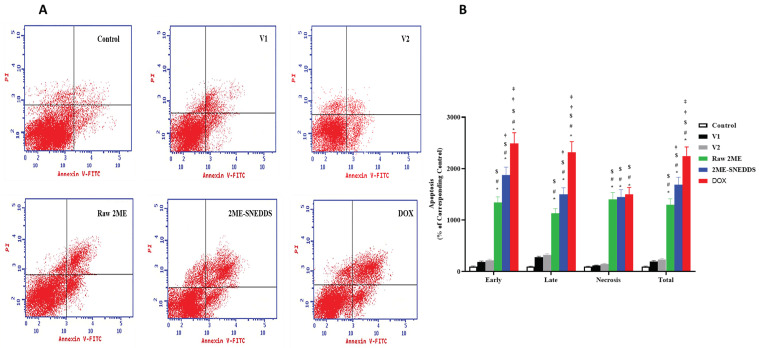
Flow cytometric analysis of early (lower left quadrant) and late apoptosis (lower right quadrant) and necrosis (upper right quadrant) using annexin V-FITC/PI double staining in MCF-7 cells following challenge with V1, V2, raw 2ME, 2ME-SNEDDS, and DOX. (**A**) Representative flow cytometric dot plots, (**B**) graphic presentation of early apoptosis, late apoptosis, necrosis, and total MCF-7 cells death. Results are presented as mean ± SD (*n* = 6). * Significantly different from control at *p* < 0.05, # significantly different from V1 at *p* < 0.05, $ significantly different from V2 at *p* < 0.05, † significantly different from raw 2ME at *p* < 0.05, ‡ significantly different from 2ME-SNEDDS at *p* < 0.05.

Treated cells were stained by annexin V-FITC/PI and analyzed with a flow cytometer to determine the percentage of cells in early and late apoptosis, and necrosis. The exposure of MCF-7 cells to raw 2ME induced early and late apoptotic cells, and necrosis (15.48%, 6.58%, and 6.05%, respectively). However, the 2ME-SNEDDS showed a significant increase in the early and late apoptotic cells, and necrosis (21.54%, 8.69%, and 6.24%, respectively). DOX exhibited a significant increase in the different stages of apoptosis (Figure 7).

### 3.7. Assessment of mRNA Expression of Bax, and Bcl-2

**Figure 8:**
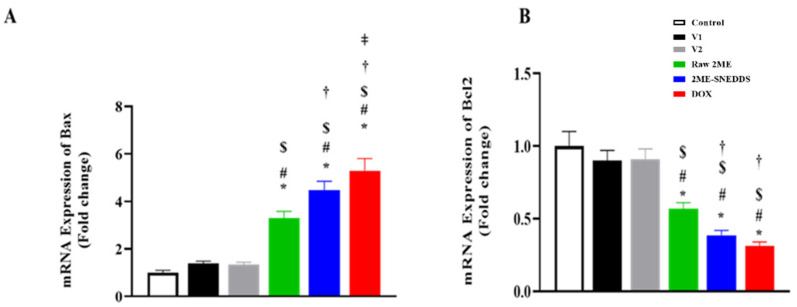
mRNA expressions of Bax (**A**), and Bcl-2 (**B**) in MCF-7 cells after incubation with V1, V2, raw 2ME, 2ME-SNEDDS, and DOX. Results are presented as mean ± SD (*n* = 6). * Significantly different from control at *p* < 0.05, # V1 at *p* < 0.05, $ significantly different from V2 at *p* < 0.05, † significantly different from raw 2ME at *p* < 0.05, ǂ significantly different from 2ME-SNEDDS at *p* < 0.05.

As demonstrated in Figure 8A, Bax mRNA expression was significantly up-regulated in MCF-7 cells after exposure to the 2ME-SNEDDS as compared to raw 2ME by 35.96%. In contrast, Bcl-2 mRNA expression was significantly down-regulated in MCF-7 cells after exposure to the 2ME-SNEDDS when compared to raw 2ME by 32.04%, (Figure 8B).

### 3.8. Assessment of Active Caspase-3 

**Figure 9:**
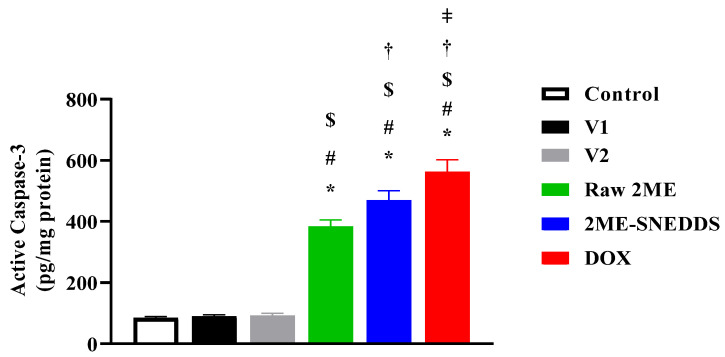
Active caspase-3 concentrations in MCF-7 cells treated with V1, V2, raw 2ME, 2ME-SNEDDS, and DOX. Results are presented as mean ± SD (*n* = 6). * Significantly different from control at *p* < 0.05, # significantly different from V1 at *p* < 0.05, $ significantly different from V2 at *p* < 0.05, † significantly different from raw 2ME at *p* < 0.05, ǂ significantly different from 2ME-SNEDDS at *p* < 0.05.

As shown in Figure 9, raw 2ME-treated cells showed an increase in the active caspase-3 concentration (384.43%) as compared to the control untreated cells. Furthermore, the 2ME-SNEDDS exhibited a significant enhancement in the active caspase-3 concentration (469.4%). Interestingly, cells challenged with the 2ME-SNEDDS exhibited a significantly higher concentration of caspase-3 as compared to the raw 2ME.

### 3.9. Effect of Raw 2ME and 2ME-SNEDDS on ROS Generation 

**Figure 10:**
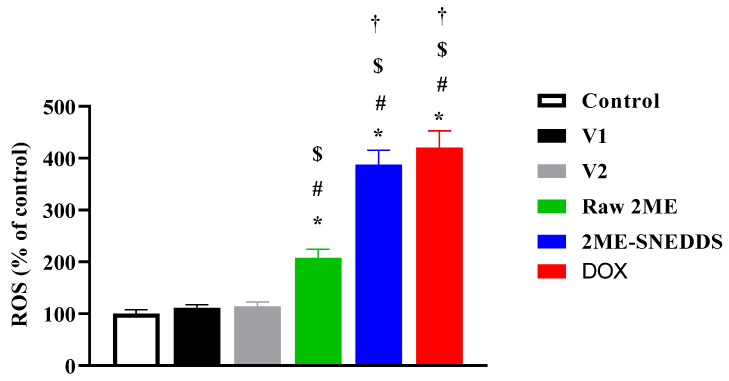
Generation of ROS in MCF-7 cells incubated with V1, V2, raw 2ME, 2ME-SNEDDS, and DOX. Results are presented as mean ± SD (*n* = 6). * Significantly different from control at *p* < 0.05, # significantly different from V1 at *p* < 0.05, $ significantly different from V2 at *p* < 0.05, † significantly different from raw 2ME at *p* < 0.05.

The data in Figure 10 indicate that cells incubated with raw 2ME increased ROS generation (207.5%) as compared to the control value, whereas the 2ME-SNEDDS had a significant potential to induce ROS generation in MCF-7 cells (387.5%). In addition, the 2ME-SNEDDS was significantly more potent in generating ROS when compared to the raw 2ME.

## 4. Discussion

Breast cancer is still one of the most common malignancies and the second leading cause of death among women worldwide [26]. 2ME is a metabolite of estrogens and possesses anti-proliferative and cytotoxic activities [27]. It has been escalated to different phases of clinical trials [9,28]. However, it suffers unfavorable pharmacokinetic characteristics and, consequently, has poor cellular permeability [14,15]. Therefore, it has been suggested to formulate 2ME in nanosuspension to enhance its delivery to tumor tissues [29]. In this study, the ability of an optimized self-nanoemulsifying drug delivery system to enhance 2ME cytotoxicity and pro-apoptotic activities was evaluated. The optimized formula had a globule size of 94.97 nm. This size is consistent with previous reports of an optimal globule size < 400 nm for solid tumors when using lipid-based delivery systems including nanoemulsifying systems [30]. Our data indicated that both the 2ME-SNEDDS and raw 2ME had significant cytotoxic effects against MCF-7 cells. Primarily, these data gain support from several studies reporting the growth inhibition of 2ME on MCF-7 cells [31,32,33]. Moreover, 2ME has been shown to sensitize breast cancer cells to doxorubicin [34], taxanes [35], and radiation [36]. Our findings also showed that the 2ME-SNEDDS exhibited more potent cytotoxicity when compared to raw 2ME. The formulation of 2ME in nanosized particles has been demonstrated to enhance its cytotoxicity in MCF-7 cells [37]. As a delivery system, the SNEDDS has been proven as effective in potentiating the cytotoxicity of several molecules in MCF-7 cells. These include curcumin [38], tamoxifen [39], docetaxel [40], quercetin [41], and sunitinib [42]. Furthermore, our findings are consistent with a recent study reporting the advantageous combination of the 2ME-SNEDDS with doxorubicin against MCF-7/ADM cells [43]. This suggests the better cellular permeability of the 2ME-SNEDDS and can be explained on the basis of the rapid internalization of its component, tween 80-stabilized oily droplets, by fluid phase pinocytosis in contrast to the raw drug that classically crosses cell membranes by passive diffusion [44].

Cyclin D1 is a key regulator of the cell cycle, mediating transition from the G1 to the S phase [45] and has been recommended as a prognostic predictive marker for breast cancer cell proliferation and metastasis [46]. Our data show that both raw 2ME and the 2ME-SNEDDS down-regulated mRNA expression cyclin D1, which provides support to the observed cell growth inhibition by 2ME. In addition, this finding is in line with other studies highlighting the ability of 2ME to down-regulate cyclin D1 in MCF-7 cells [47] and other cells [48,49]. To further substantiate the cytotoxicity of 2ME-SNEDDS, its impact on cell cycle phases was evaluated. Both the 2ME-SNEDDS and raw 2ME caused an accumulation of MCF-7 cells in the G0/G1 and G2/M phases. This is consistent with previous studies that indicated the ability of 2ME and/or its combination with other drugs to enhance cancer cells in G0/G1 [34,50]. Moreover, the observed down-regulation of cyclin D1 lends support to the increased cell population in the G0/G1 phase. Furthermore, the observed increase in the percentage of cells in the G2/M phase is consistent with the known tubulin inhibiting activity of 2ME as well as those data reported in the phases in the literature [51,52,53]. An additional study showed that 2ME causes cell cycle arrest in both the G0/G1 and G2/M phases depending on its concentration. This suggested a dual mechanism of action of 2ME; tubulin polymerization inhibition and a tubulin-independent one [54]. In this regards, the effect of the 2ME-SNEDDS was more pronounced on the pre-G1 fraction, which indicates apoptotic cell death as suggested in previous studies [55]. The finding gains support from an earlier study that showed that the exposure of MCF-7 cells to 2ME was associated with an increased percentage of sub-G population [56]. Annexin V staining confirmed these data and indicated a greater apoptotic-enhancing activity of 2ME in the prepared 2ME-SNEDDS compared with the raw 2ME. Early and late apoptotic death, as well as total cell death, were all included. This is consistent with previous reports on 2ME’s pro-apoptotic activities in MCF-7 breast cancer cells [37,56,57,58]. It also increases the apoptosis caused by chemotherapeutic agents such as docetaxel [59]. 2ME-induced apoptosis has been suggested to occur via both the mitochondrial pathway [60] and the up-regulation of death receptors [61]. Since cells exposed to both vehicles used in this study did not show an appreciable potentiation of apoptotic cells, it can be suggested that the observed activity is attributable to enhanced 2ME cell penetration by the SNEDDS formula. 

Bax and Bcl-2 are respective pro-apoptotic and anti-apoptotic proteins that play a pivotal role in controlling apoptosis of neoplastic mammary cells [62]. Therefore, their mRNA expression was assessed in this study. Our results indicated that 2ME-SNEDDS significantly up-regulated the expression of Bax. On the other hand, raw 2ME and the 2ME SNEDDS up-regulated the expression of Bcl-2. These data further support the detected pro-apoptotic activities of the 2ME-SNEDDSS and are consistent with previous reports in cancer cells [63,64,65] and benign hyperplasia [18,66]. In cells undergoing apoptosis, the activation of caspase-3, a crucial enzyme in the execution of the apoptotic pathway, has been extensively used as a biomarker of cell death [67]. In the current study, the 2ME-SNEDDS significantly enhanced caspase-3 concentration in MCF-7 cells. This further supports the detected pro-apoptotic effects of the 2ME-SNEDDS throughout the study. This is line with the reported up-regulation and activation of caspase-3 by 2ME [37,68,69]. Furthermore, it was observed that 2ME-SNEDDS is more potent in enhancing cleaved caspase-3 in MCF-7 cells. This is in harmony with previous reports showing the additive apoptosis potentiating properties of the SNEDDS in different cells and tissues [21,70]. Considering the neutral effects of both vehicles used in this study with regard to caspase-3 concentration, it can be deduced that caspase-3 activation properties are credited to 2ME itself and not to the coating SNEDDS. Further to this, it was observed that the apoptotic effects of raw 2ME and the 2ME-SNEDDS were associated with an increased production of reactive oxygen species. The data further strengthen the observed pro-apoptotic activities of 2ME. Additionally, these data are in line with the reports that suggest the generation of oxygen species as a mediator of 2ME cytotoxic and pro-apoptotic activities [71,72,73,74]. 

## 5. Conclusions

Preparation and optimization using mixture design to prepare 2ME in the form of a SNEDDS exhibited nanosized vesicles with an acceptable size distribution and improved 2ME solubility and release.

In the present study, a 2ME-SNEDDS formula was prepared using mixture design and was optimized with respect to particle size. The optimized formula showed acceptable solubility and release. Compared to raw 2ME, the optimized formula exhibited enhanced anti-proliferative activity in MCF-7 breast cancer cells as evidenced by the down-regulation of cyclin D1 and inhibition of cell cycle progression. This was associated with a significant increase in apoptotic activity as shown by the modulation of annexin V staining, Bax and Bcl-2 mRNA expression, and caspase-3 concentration in favor of apoptosis. In addition, the incubation of cells with the optimized formula resulted in an increased formation of ROS. Collectively, the optimized 2ME-SNEDDS formula possesses enhanced anti-proliferative and pro-apoptotic activity against MCF-7 cells. This is mediated by, at least partially, the stimulation of ROS generation.

## Figures and Tables

**Table 1:** Experimental runs of 2ME-SNEDDS and their measured droplet size.

Run #	Mixture Components’ Proportions *			Droplet Size (nm)
	Cumin Oil; X_1_	Tween 80; X_2_	PG; X_3_	
1	30	35	35	428
2	20	40	40	313
3	21.5	31.5	47	660.4
4	30	40	30	1268
5	30	40	30	1268
6	30	25	45	283
7	25	40	35	261.9
8	15	35	50	409
9	15	35	50	410
10	25	25	50	659
11	30	25	45	280.7
12	26.5	32.75	40.75	345.9
13	25	25	50	657
14	15	40	45	240
15	19	36.5	44.5	278.5
16	15	40	45	455.3

Abbreviations: PG, propylene glycol. * The proportions of each run are summed up to 100.

**Table 2:** Primer sequence.

mRNA Target	Accession Number	Primer Direction	Sequence (5’->3’)
Bax	NM_001291430.2	Forward	ATGGACGGGTCCGGGGAG
		Reverse	ATCCAGCCCAACAGCCGC
Bcl-2	XM_047437734.1	Forward	AAGCCGGCGACGACTTCT
		Reverse	GGTGCCGGTTCAGGTACTCA
Cyclin D1 (CCND1)	NM_053056.3	Forward	CCGTCCATGCGGAAGATC
		Reverse	GAAGACCTCCTCCTCGCACT
		Reverse	CCCTCAGAGAATCGCCAGTACT
β-actin	NM_001101.5	Forward	ATCGTGGGGCGCCCCAGGCAC
		Reverse	CTCCTTAATGTCACGCACGATTTC

**Table 3:** Model fit statistics of 2ME-SNEDDS according to special quartic model.

Sequential *p*-Value	Lack of Fit *p*-Value	SD	R^2^	Adjusted R^2^	Predicted R^2^	PRESS
0.0036	0.4628	104.55	0.9515	0.8961	0.7341	4.195 × 10^5^

Abbreviations: systems; R^2^, multiple correlation coefficient; PRESS, predicted residual error sum of squares.

**Table 4:** Analysis of variance output for the effect of mixture components on the droplet size of 2ME-SNEDDS.

Source	Sum of Squares	Degrees of Freedom	Mean Square	F-Value	*p*-Value
Model	1.566 × 10^6^	8	1.958 × 10^5^	37.01	<0.0001
Linear Mixture	4.285 × 10^5^	2	2.142 × 10^5^	40.51	0.0001
X_1_X_2_	20,982.46	1	20,982.46	3.97	0.0867
X_1_X_3_	27,114.11	1	27,114.11	5.13	0.0580
X_2_X_3_	39,600.87	1	39,600.87	7.49	0.0291
X_1_^2^X_2_X_3_	21,375.14	1	21,375.14	4.04	0.0843
X_1_X_2_^2^X_3_	15,821.30	1	15,821.30	2.99	0.1273
X_1_X_2_X_3_^2^	48,768.06	1	48,768.06	9.22	0.0189
Residual	37,024.11	7	5289.16		
Lack of Fit	13,841.92	2	6920.96	1.49	0.3102
Pure Error	23,182.19	5	4636.44		
Cor Total	1.603 × 10^6^	15			

## Data Availability

Data are contained in the article.
