# Correction: Al-Qahtani et al. Self-Nanoemulsifying Drug Delivery System of 2-Methoxyestradiol Exhibits Enhanced Anti-Proliferative and Pro-Apoptotic Activities in MCF-7 Breast Cancer Cells. *Life* 2022, *12,* 1369

**DOI:** 10.3390/life13061343

**Published:** 2023-06-08

**Authors:** Salwa D. Al-Qahtani, Hawazen H. Bin-Melaih, Eman M. Atiya, Usama A. Fahmy, Lenah S. Binmahfouz, Thikryat Neamatallah, Fahad A. Al-Abbasi, Ashraf B. Abdel-Naim

**Affiliations:** 1 Department of Medical Laboratory Sciences, Faculty of Applied Medical Science, Majmaah University, Majmaah 11952, Saudi Arabia; 2 Department of Biochemistry, Faculty of Science, King Abdulaziz University, Jeddah 21589, Saudi Arabia; 3 Department of Biological Sciences, Faculty of Sciences, King Abdulaziz University, Jeddah 21589, Saudi Arabia; 4 Department of Pharmaceutics, Faculty of Pharmacy, King Abdulaziz University, Jeddah 21589, Saudi Arabia; 5 Department of Pharmacology and Toxicology, Faculty of Pharmacy, King Abdulaziz University, Jeddah 21589, Saudi Arabia

## Error in Figure

In the original publication [1], there was a mistake in Figure 6A and Figure 7A as published. One sub-image in the replicates of negative control V1 was uploaded instead of V2. The corrected Figure 6 and Figure 7 appear below. The authors state that the scientific conclusions are unaffected. This correction was approved by the Academic Editor. The original publication has also been updated.

## Figures and Tables

**Figure 6:**
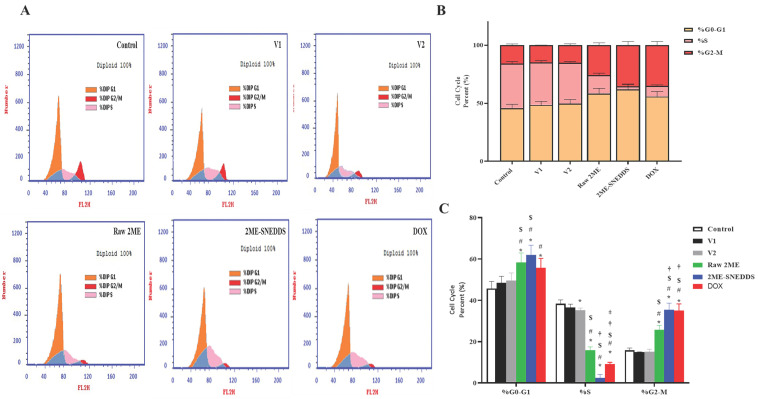
Flow cytometric analysis of cell cycle distribution of PI-stained MCF-7 cells. (**A**) Representative flow cytometry histograms after treatment with V1, V2, raw 2ME, 2ME-SNEDDS, and DOX. (**B**) Cumulative bar chart showing the percentage of cells in the G0/G1, S, and G2/M phases of the cell cycle. (**C**) Graphic presentation of the percentages of cell cycle phases. Results are presented as mean ± SD (*n* = 6). * Significantly different from control at *p* < 0.05, # significantly different from V1 at *p* < 0.05, $ significantly different from V2 at *p* < 0.05, † significantly different from raw 2ME at *p* < 0.05, ‡ significantly different from 2ME-SNEDDS at *p* < 0.05.

**Figure 7:**
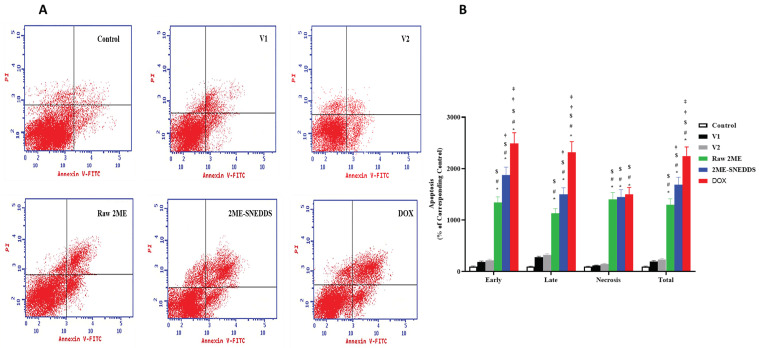
Flow cytometric analysis of early (lower left quadrant) and late apoptosis (lower right quadrant) and necrosis (upper right quadrant) using annexin V-FITC/PI double staining in MCF-7 cells following challenge with V1, V2, raw 2ME, 2ME-SNEDDS, and DOX. (**A**) Representative flow cytometric dot plots, (**B**) graphic presentation of early apoptosis, late apoptosis, necrosis, and total MCF-7 cells death. Results are presented as mean ± SD (*n* = 6). * Significantly different from control at *p* < 0.05, # significantly different from V1 at *p* < 0.05, $ significantly different from V2 at *p* < 0.05, † significantly different from raw 2ME at *p* < 0.05, ‡ significantly different from 2ME-SNEDDS at *p* < 0.05.
